# Study on the Swimming Behavior of Black Carp (*Mylopharyngodon piceus*) and Silver Carp (*Hypophthalmichthys molitrix*) in Early Developmental Stage

**DOI:** 10.3390/ani14223221

**Published:** 2024-11-10

**Authors:** Junjun Tan, Xueqin Zhu, Junjian Sun, Yuanyang Wang, Hongqing Zhang, Senfan Ke, Giri Raj Kattel, Xiaotao Shi

**Affiliations:** 1 Hubei International Science and Technology Cooperation Base of Fish Passage, China Three Gorges University, Yichang 443002, China; tanjunjun52@163.com (J.T.); zhu_xq9938@163.com (X.Z.); sun_jj1@163.com (J.S.); wangyuanyang911@163.com (Y.W.); ksfctgu@163.com (S.K.); 2 College of Hydraulic and Environmental Engineering, China Three Gorges University, Yichang 443002, China; 3 Zhejiang Institute of Hydraulics & Estuary (Zhejiang Institute of Marine Planning and Design), Hangzhou 310020, China; zhanghangqing8@126.com; 4 Department of Infrastructure Engineering, The University of Melbourne, Victoria 3010, Australia; giri.kattel@unimelb.edu.au; 5 Department of Hydraulic Engineering, Tsinghua University, Beijing 100084, China

**Keywords:** swimming patterns, carp, hydrodynamic conditions, water velocity

## Abstract

Fish need to make different responses to different water flow conditions, as a variety of fish utilize or overcome the water flow while fish are in the water. This study aims to analyze the response of two types of carp (black carp and silver carp) to water flow in different body lengths (2.0–10.0 cm) in early developmental stages in an open channel. By analyzing the water velocity and fish swimming behavior, swimming against the water flow was the most frequent swimming behavior, and the frequencies of this behavior increased with the increased body length of fish. The results demonstrated that the detectable water velocity of two carp species of different body lengths remained in a certain range (0.020–0.060 m/s). Black carp and silver carp can swim against the water flow of 0.295–0.790 m/s and 0.245–0.825 m/s, respectively. The results of this study can provide invaluable data for engineers and biologists to protect fish in the early developmental stage and restore the ecological habitats in the regulated river systems.

## Simple Summary

The early developmental stage is an important stage in the entire life cycle of fish. It is critical to understand the impact of water velocity on fish. In this work, we studied black carp and silver carp of different body lengths (2.0–10.0 cm) in an open channel by analyzing the relationship between water velocity and fish behaviors in early developmental stages. The results showed that two types of carp can detect water velocity ranged of 0.020–0.060 m/s. Among the four swimming patterns, the most common behavior was swimming against the water flow and moving downstream. The frequencies of swimming against the water flow increased with the increased body length and water velocity. Our findings provide protection for the fish in the early developmental stage.

## 1. Introduction

The construction of anthropogenic barriers such as dams, culverts, and other water structures has altered natural flow regimes; water flow regimes are a critical factor for the maintenance of fish habitat, their life history, and reproduction under natural conditions [1,2]. Changes in water flow regimes can affect hydrodynamic conditions in rivers. Fish are subjected to hydrodynamic factors during their migratory movements in rivers [3]. Conditions of flows in riverine systems may be perceived by fish through their hydrodynamic sensory system [4] and used as cues driving fish behavioral responses. Hence, to evaluate the sensitivity of fish to water flow, we measured the velocity of the fish to detect water flow.

The hydrodynamic sensory system of fish not only detects water flow but also allows fish to orient their swimming with respect to hydrodynamic cues [5]. Fish swimming behavior is one of the main life movements of fish, which is a vital content in the fish behavioral research field [6]. This research is concerned with the direction of swimming in fish, such as moving upstream, downstream, and fallback [7,8]. However, many studies suggested that the swimming behavior of fish is intricately linked to water velocity; this can either increase [9] or decrease [10] the energy consumption of fish movement and create instability in swimming behavior [11,12]. Accordingly, we believe it is necessary to study the effect of water velocity on fish behavior.

Fish in the early developmental stage refers to the period from embryonic, larval stage, and juvenile stages [13,14]. Mortality rates of fish are generally high, and water flow is crucial for fish species’ lives in early developmental stages [15,16]. Four major Chinese Carp (FMCC), including black carp *Mylopharyngodon piceus* (Richardson, 1846), grass carp *Ctenopharyngodon idella* (Valenciennes, 1844), silver carp *Hypophthalmichthys molitrix* (Valenciennes, 1844), and bighead carp *Hypophthalmichthys nobilis* (Richardson, 1845), are commercially valuable species in the Yangtze River [17,18], as well as their migration routes are between rivers and lakes [19,20]. Black carp and silver carp are two typical types of carp that are usually important research objectives. Thus, this study is mainly related to black carp and silver carp [21].

Currently, the studies on the two types of carp (black carp and silver carp) are mainly related to fish swimming abilities or performance [22,23]. The body length of the fish and the swimming speed are generally correlated; many studies have used body length as an important indicator [24,25]. However, the ability to detect water flow and swim against water flow with two types of carp in different body lengths in early developmental stages still needs to be studied.

This study aims to analyze the response of two types of carp (black carp and silver carp) to water flow in different body lengths in early developmental stages in an open channel. Therefore, the specific objectives of this paper were to (1) identify the ability of the fish to detect water flow and (2) quantify the ability to swim against water flow. The results provide valuable data for engineers and biologists to protect fish resources and restore ecological habitat.

## 2. Materials and Methods

### 2.1. Test Apparatus

**Figure 1:**
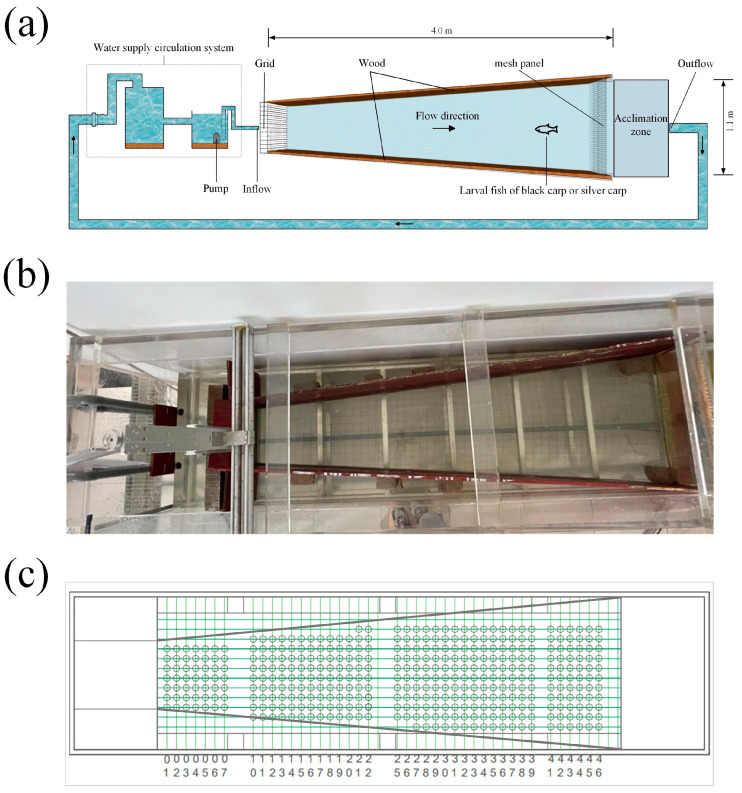
(**a**) Schematic diagram of the experimental setup; (**b**) the experimental apparatus; (**c**) array of measurement points in each horizontal plane.

The experiment was conducted in a trapezoidal open channel with a dimension of 4.00 m (length) × 1.10 m (top width) × 0.20 m (bottom width) (Figure 1b). A water supply circulation system including a pump (rated discharge: 0.05 m^3^/s), a plastic water pipe, and one large water tank (diameter × height: 2.0 m × 1.8 m) was used to circulate water in the channel (Figure 1a). During the experiment, the range of water velocity in the channel was 0.1–0.5 m/s. To obtain fish swimming movements in 2-D (*x*- and *y*-axes), a video camera (640 × 480 pixels, 25 fps) was positioned above the channel, which can cover the entire test zone. Logger Pro software (Vernier Software and Technology, version 3.16.2) was used to obtain fish movement behavior, including fish swimming speed and transit stayed position, by analyzing the video data.

### 2.2. Hydraulics

Experimental conditions are provided in Table 1. An Acoustic Doppler Velocimeter (ADV, manufactured by Nortek, Norway) with a data collection frequency of 200 Hz and a sampling time of 30 s at each point was used to measure the water velocity in the channel. Measurements were taken at the middle of the horizontal plane parallel to the flume bottom, namely *z* = 0.5 h (where h represents the average water depth in the pool). Flow velocities were measured at a total of 102 points in the horizontal plane *z* = 0.5 h. We selected 17 cross-sections that were measured, six data points per cross-section (Figure 1c). WinADV software (version 2.0.0024) was used to obtain data [26].

### 2.3. Experimental Fish

All experimental fish, including black carp (*N* = 79) and silver carp (*N* = 86), with the range of total length (2.0–10.0 cm) and body weight (0.8–16.2 g) (the detailed description of the experimental fish can be seen in Table 1). The experimental fish were obtained from a hatchery near Qingjiang River, Hubei, China. The water in the hatchery is from the Qingjiang River and was used to simulate the water flow environment in the river as much as possible. All the individuals were collected and transported to the Technology Cooperation Base of Fish Passage, China Three Gorges University, and kept in an aerated tank (diameter × height: 1.8 m × 0.5 m; water depth: 0.3 m) before the experiment started. To recover from transport and handling stress, fish were kept in the tanks for at least 3 days before the experiment. The fish were fed pond sticks (Tetra GmbH) until 24 h prior to experimentation. Constant recirculation water exchange was used to stabilize the water temperature. The water temperature was stabilized (mean ± SD = 20.1 ± 0.7 °C) during the experimental period.

### 2.4. Experimental Methods

For one test, one fish was randomly selected and placed in the acclimation zone near the channel. After a 10 min acclimation in the test channel, the individual fish was allowed to volitionally move in the channel after removing the mesh panel. The test fish were continuously monitored for swimming behavior in response to water flow, and their movements were recorded by the video recording system. The ability to detect water flow is the minimum swimming ability at which a fish responds to water flow [27,28]. The ability to detect water flow for two types of carp (black carp and silver carp) was determined by the fish head facing the direction of the water flow and its tail swinging uniformly in the open channel when the fish was swimming in the water flow. When the direction of the fish’s head and the direction of the water flow are opposite, the test fish will change its movement behavior from active swimming to against water flow; fish are considered exhausted when they lose the ability to swim against the water flow and are swept to the end of the experimental device. Thus, the test for the ability of fish to swim against the water flow was terminated. After these, fish may exhibit behaviors such as holding the station, moving downstream, and fallback. One fish was monitored within 1 h. One tested fish was just used one time. After the test, all the tested fish were released back into the river.

### 2.5. Data Analysis

After the experiment, the data were collected and analyzed in all experiments based on the video data and monitoring. Statistical analysis was conducted using the SPSS software (IBM SPSS Statistics 26.0); the detected water velocity and the corresponding body length of the test fish were obtained; data were presented as the mean ± standard deviation (S.D.); the ability to swim against water flow velocities within a certain body length range was used to calculate the average swimming velocity against water flow in this specific body length range. In addition, the Origin 2021 software (version 9.850212) package was used to process individual figures.

## 3. Results

### 3.1. Swimming Behavior of Fish

**Figure 2:**
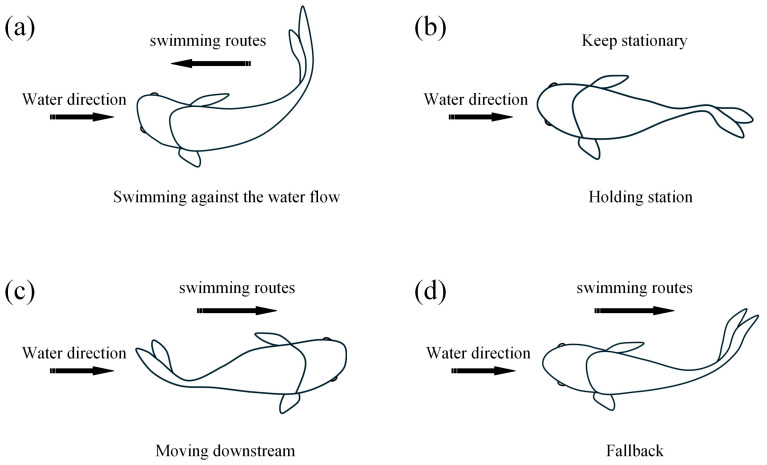
Schematic of the four swimming behaviors. (**a**) swimming against the water flow of the fish; (**b**) holding station of fish; (**c**) moving downstream of fish; (**d**) fallback of fish.

According to the fish behaviors observed by the camera throughout the experiment, both black carp and silver carp showed five swimming behavior patterns in the swimming process, i.e., detecting water flow, swimming against the water flow, holding station, moving downstream, and fallback. The behavior of detecting water flow is a sign that the test fish starts to swim against the water flow. When the head of the test fish is facing the direction of the water flow and the tail swing changes from uneven to uniform, it is the behavior of detecting water flow, and the water velocity at this time is the velocity of detecting water flow. Then, the tail of the test fish swam uniformly and swam against the water flow, and the direction of the fish’s head was always opposite to the direction of the water flow. When the fish swims against the water flow to the point that it cannot swim forward anymore, it holds station for a few seconds, and then there are two kinds of behaviors: moving downstream or falling back in the bottom stream (Figure 2).

It was observed that detect water flow appeared at the beginning of the experiment, and we obtained the velocity of detected water flow of both fish and explored the relationship between body length and the ability to detect water velocity. At the same time, we counted the frequencies of the four behaviors: swim against the water flow, hold station, move downstream, and fallback (Table 2). It is obvious that the frequencies of swimming against the water flow and moving downstream for silver carp were significantly higher than those for black carp across all body length ranges. In addition, swimming against the water flow and moving downstream were the most common swimming behaviors in the test.

### 3.2. The Ability to Detect Water Flow for Two Types of Carp in Their Early Developmental Stage

**Figure 3:**
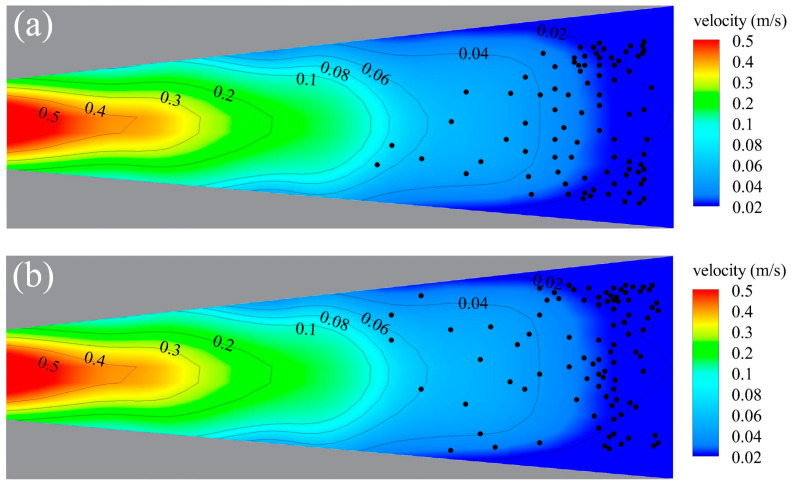
Simulation results of the flow field in the study zone. Dots show the distribution of detected water velocities among (**a**) black carp and (**b**) silver carp in the early developmental stage.

In this study, a total of 95 black carp and 101 silver carp in the early developmental stage were tested, with both body lengths ranging from 2.0 to 10.0 cm (Table 1). The water velocities were evenly spread out in the open channel. By combining the water flow field and the location positions of the test fish in the open channel, it was found that the positions of the tested fish that can detect water flow were mainly in a certain area, with a water velocity of 0.020–0.060 m/s (Figure 3a). Specifically, the positions in black carp that can detect water velocities were concentrated in the range of 0.020–0.040 m/s on both sides of the channel, while the positions of silver carp that can detect water velocities were concentrated around 0.030–0.060 m/s (Figure 3b).

### 3.3. The Ability to Swim Against the Water Flow for Two Types of Carp in Their Early Developmental Stage

**Figure 4:**
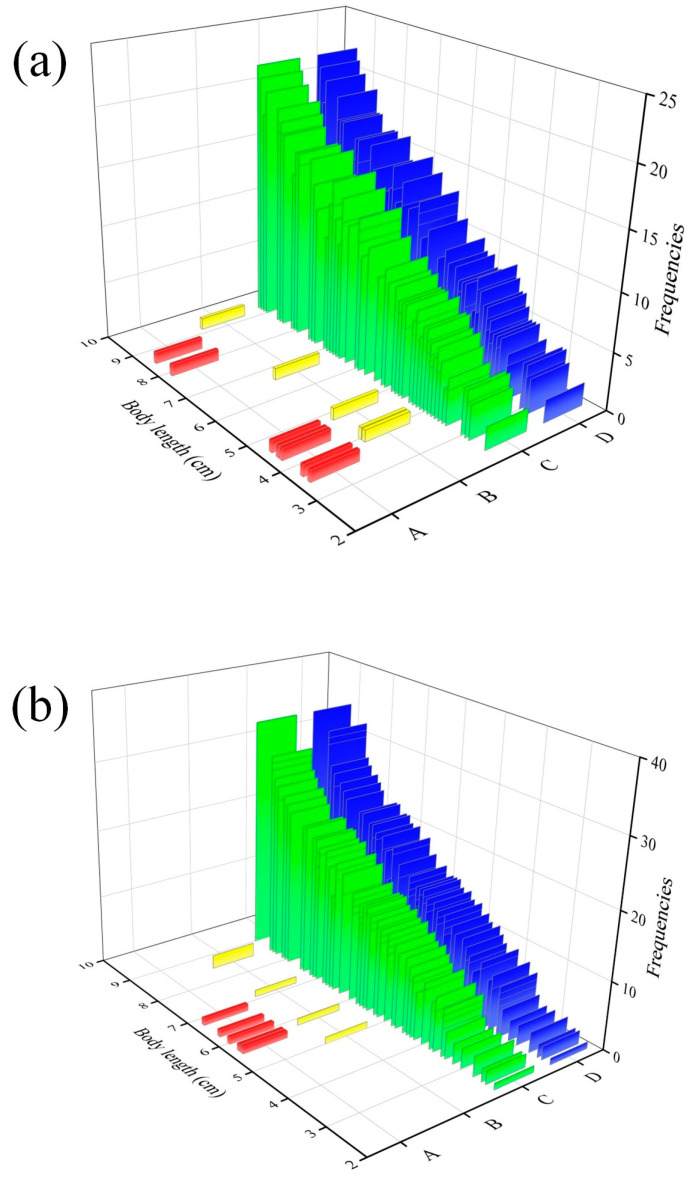
Relationships between moving behavior (A: fallback (red color); B: holding station (yellow color); C: swimming against the water flow (green color); and D: moving downstream (blue color)) and body size of (**a**) black carp and (**b**) silver carp in the early developmental stage. Frequencies indicate the number of each swimming behavior occurring for target fish.

It was observed that the movement behavior of the test fish was mostly swimming against the water flow (i.e., moving upstream) and moving downstream, sometimes holding station or fallback (i.e., the head of the fish is moving in the opposite direction of the flow and downstream) in the process of movement (Figure 4). The frequencies of swimming against the water flow, moving downstream, holding station, and fallback for black carp and silver carp with different body lengths in the water flow were obtained by analyzing the video monitoring data, as shown in Table 2. We further analyzed the relationship between fish body length and different swimming behaviors, and it was demonstrated that the frequencies of swimming against the water flow and moving downstream increased with the increased body length of black carp and silver carp (Figure 4). However, the frequencies of holding station and fallback for two types of carp did not regularly vary.

The body length of black carp was nearly linear to the frequencies of swimming against the water flow and moving downstream; silver carp had a similar situation. The difference is that the overall growth trend of black carp is slow (Figure 4a), while the growth trend of silver carp from 7.0 to 10.0 cm is rapid (Figure 4b). In addition, the overall frequencies of silver carp were higher than those of black carp, and silver carp were more active than black carp. There was no significant difference between the two for the frequencies of holding station and fallback.

**Figure 5:**
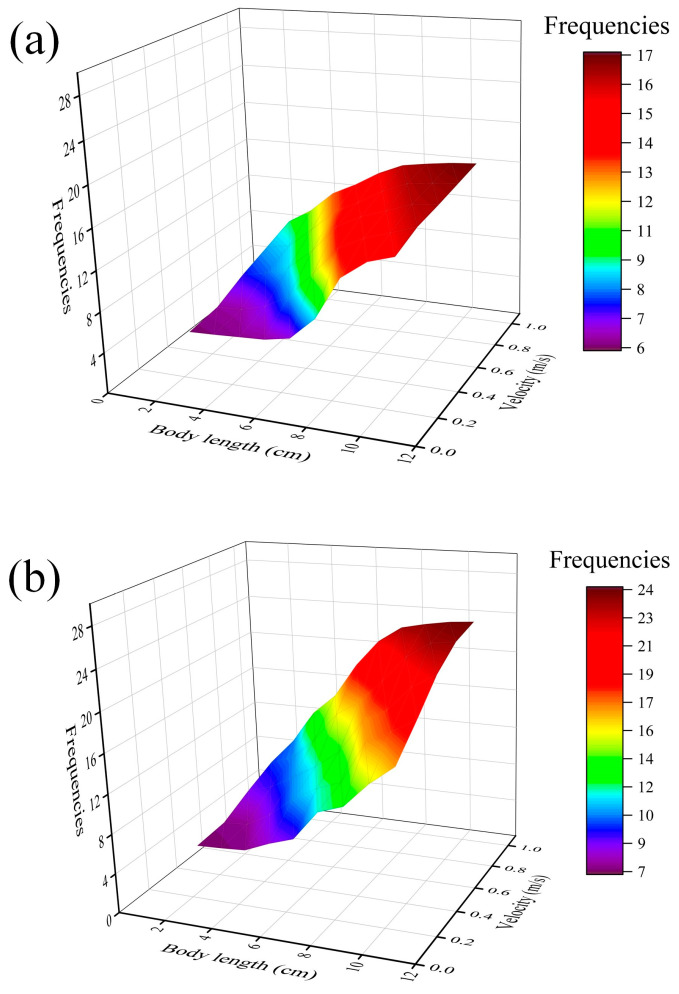
Relationships between swim against the flow frequencies, velocity, and different body lengths of (**a**) black carp and (**b**) silver carp in the early developmental stage. (Note: Velocity indicates the target fish swim against the water velocity; frequencies indicate the average of the frequencies they swim against the water flow and move downstream.).

**Figure 6:**
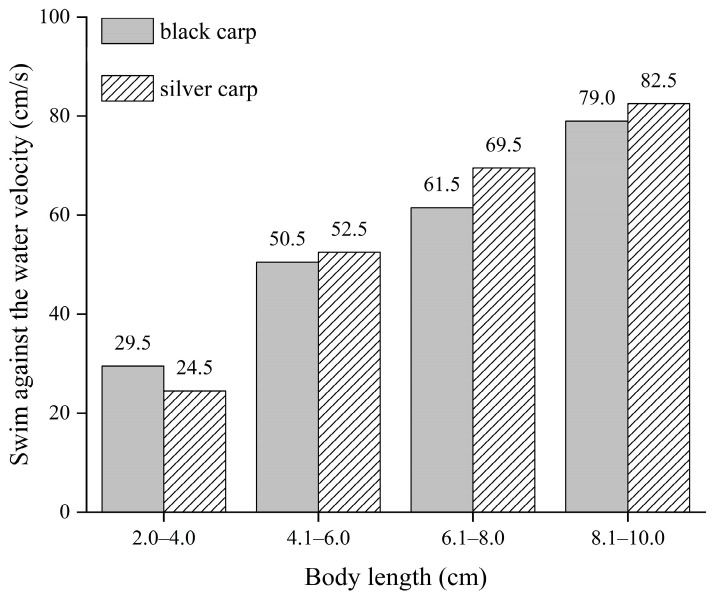
Swimming against the water flow with different body lengths of black carp and silver carp in the early developmental stage. The number above each column indicates the target fish’s swimming velocity against the water flow.

Swimming against the water flow frequencies increases with body length and is correlated with water velocity. We found similar trends when comparing water velocity and swimming against the water flow frequencies with body length. The data indicated that the ability to swim against the water flow increased with the increased body length of the test fish (Figure 5). In contrast, we found that silver carp were fastest when swimming against the water flow with increasing body length, which implied that the ability of silver carp to swim against the water flow was greater than that of black carp. By coupling the water velocity with the different body lengths of the tested fish, the velocity range for fish swimming against water flow was 0.295–0.790 m/s for black carp and 0.245–0.825 m/s for silver carp in their early developmental stage (Figure 6). Furthermore, black carp with 2.0–4.0 cm in body length had greater capability to swim against the water flow than silver carp. However, black carp with 4.0–10.0 cm in body length had a smaller capability to swim against the water flow than silver carp in all tests.

## 4. Discussion

In this study, the relationship between water flow and fish swimming behavior with different body lengths (2.0–10.0 cm) of black carp and silver carp in the early developmental stage was analyzed. Fish usually exhibit sensitivity to water velocity [29], and suitable water velocity can trigger the movement of fish [30,31]. In this paper, coupling the velocity of detecting water velocity and different body lengths of test fish can be used to assess the ability of fish to detect water flow. It was concluded that the detected flow velocities of black carp and silver carp were both 0.020–0.060 m/s in the early developmental stage. Although there was no major increase or decrease trend with the variation in body length, which is consistent with the results of the studies conducted by Bai et al. [32] and Li et al. [33], the location of the detected water flow was concentrated in a certain area within the test area for both species (Figure 3).

Fish can instinctively swim against the water flow and detect water flow by means of lateral line receptors, and swim against the water flow and hold station with the increased water velocity [34,35]. The fish in the early developmental stage (i.e., embryonic, larval stage, and juvenile stage) is severely impacted by water flow compared to adult fish. The swimming behavior of adult fish was characterized by free-swimming in the water with brief bouts of occasional tail-bracing arising prior to fatigue [36]. However, the fish in the early developmental stage did not present a similar situation. In our study, there were four swimming behaviors: swimming against the water flow, holding a station, moving downstream, and falling back. The most common swimming behavior of the two types of carp was swimming against the water flow, which is consistent with the rheotaxis of fish. In addition, as body length increased, we found that both two types of carp exhibited similar patterns when they swam against the water flow and moved downstream in the early developmental stage. During the whole process of the experiment, the frequencies of both species fallback and moving downstream were less likely than that of swimming against the water flow and downstream movement (Figure 4). The reason may be that the higher energy loss during swimming against the water flow causes most test fish to move downstream [37]. In comparison, silver carp demonstrated a higher swimming against the water flow and a greater frequency of moving downstream than black carp. This suggests that silver carp tend to swim more actively.

The body length was one of the main factors influencing the ability of fish to swim [38,39]. In this study, after analyzing and comparing the body length and the frequencies of swimming against the water flow of the test fish during the experiment (Figure 5), the results showed that as water flow velocity and body length increased, the ability of fish to swim against the flow increased, which is consistent with the results of other research [40,41]. However, the differences in the two types of carp’s swimming performance considering other factors such as turbulent energy, eddies, and temperature [42,43,44] still need to be further explored. Further work should be conducted to more comprehensively analyze carp’s swimming behavior in the early developmental stage.

## 5. Conclusions

This paper explored the response relationship between water velocity and fish swimming behavior with different body lengths (2.0–10.0 cm) of black carp and silver carp in the early developmental stage in an open channel. The results showed that the most common swimming behavior of two types of carp was swimming against the water flow, and the frequencies of this behavior increased with the increase in body length. Two types of carp can detect water velocity ranging from 0.020 to 0.060 m/s. Black carp can swim against the water flow of 0.295–0.790 m/s, and silver carp can swim against the water flow of 0.245–0.825 m/s. The results can provide valuable data for engineers and biologists to protect fish resources.

## Figures and Tables

**Table 1:** Morphological parameters and detect the water velocity of target fish.

Species	*N*	Body Length (cm)	Body Weight (g)	Detected Water Velocity (m/s)
black carp	38	2.0–5.0	1.0–5.2	0.020–0.060
	41	5.1–10.0	5.5–16.2	
silver carp	38	2.0–5.0	0.8–4.8	0.020–0.060
	48	5.1–10.0	5.0–15.0	

Note: *N* indicates the number of experimental fish.

**Table 2:** The frequencies of swimming against the water flow, moving downstream, and fallback target fish.

Swimming Behavior	Body Length (cm)	Frequencies	
		Black Carp	Silver Carp
Swimming against the water flow	2.0–4.0	5 ± 1.3	6 ± 2.8
	4.1–6.0	9 ± 2.2	12 ± 2.3
	6.1–8.0	13 ± 2.0	19 ± 2.6
	8.1–10.0	18 ± 2.4	29 ± 2.8
Holding station	2.0–4.0	2	0
	4.1–6.0	2	3
	6.1–8.0	1	0
	8.1–10.0	1	2
Moving downstream	2.0–4.0	4 ± 1.4	6 ± 2.8
	4.1–6.0	9 ± 2.2	12 ± 2.2
	6.1–8.0	12 ± 2.3	19 ± 2.7
	8.1–10.0	18 ± 2.5	29 ± 3.1
Fallback	2.0–4.0	2	0
	4.1–6.0	3	3
	6.1–8.0	1	2
	8.1–10.0	1	0

Note: Frequencies indicate the average frequencies of black carp and silver carp at a given body length range, and because the frequencies of holding station and fallback were too low, we aggregated the total frequencies at a given body length range.

## Data Availability

The data presented in this study are available on request from the corresponding author.
